# Deciphering the Pharmacological Potentials of Methanol Extract of *Sterculia foetida* Seeds Using Experimental and Computational Approaches

**DOI:** 10.1155/2022/3403086

**Published:** 2022-04-23

**Authors:** Najmul Alam, Naureen Banu, Nobi ul Alam, Umme Ruman, Zidan Khan, Md. Arfin Ibn Aziz, Niloy Barua, Farhana Jahan Chy, Afrin Jahan, Tamalika Sarker Juthy, Mohamed A. Nassan, Salah M. El-Bahy, Amany Abdel-Rahman Mohamed, Mohammed Aktar Sayeed, Talha Bin Emran

**Affiliations:** ^1^Department of Pharmacy, International Islamic University Chittagong, Chittagong 4318, Bangladesh; ^2^Drug Discovery, GUSTO A Research Group, Chittagong 4203, Bangladesh; ^3^Department of Microbiology, Friedrich Schiller University Jena, Fürstengraben 1, Jena 07743, Germany; ^4^Department of Pharmacy, Jagannath University, Chittaranjan Ave, Dhaka 1100, Bangladesh; ^5^Department of Clinical Laboratory Sciences, Turabah University College, Taif University, P.O. Box 11099, Taif 21944, Saudi Arabia; ^6^Department of Chemistry, Turabah University College, Taif University, P.O. Box 11099, Taif 21944, Saudi Arabia; ^7^Department of Forensic Medicine and Toxicology, Zagazig University, Zagazig 44511, Egypt; ^8^Department of Pharmacy, BGC Trust University Bangladesh, Chittagong 4381, Bangladesh

## Abstract

The edible herb *Sterculia foetida* L. has potential nutraceutical and medicinal effects. The present study is performed to assess the possible antidiabetic, neuropharmacological, and antidiarrheal activity of the methanolic extract of *S*. *foetida* seeds (MESF) through *in vitro*, *in vivo*, and *in silico* approaches. When compared to standard acarbose, the results of the antidiabetic study provided strong proof that the glucose level in the MESF was gradually decreased by inhibiting the function of *α*-amylase enzymes. The sedative potential of MESF (200 and 400 mg/kg) was determined by employing open field, hole cross, and thiopental sodium-induced sleeping time tests, which revealed significant reductions in locomotor performance and increased sleep duration following MESF treatment. In addition, mice treated with MESF exhibited superior exploration during elevated plus maze and hole board tests. MESF also showed good antidiarrheal activity in castor oil-induced diarrhea and intestinal motility tests. Previously isolated compounds (captan, 1-azuleneethanol, acetate, and tetraconazole) exhibited good binding affinity in docking studies and drug-likeliness properties in absorption, distribution, metabolism, excretion/toxicity (ADME/T), and toxicological studies. Collectively, these results indicate the bioactivity of *S*. *foetida*, which represents a potential candidate in the food and pharmaceutical industries.

## 1. Introduction

Plants have long been used by humans to cure various diseases; however, modern medicines have trended away from the utilization of plants to prioritize the utilization of laboratory-produced agents. However, components derived from plants tend to display distinct structural diversity and reduced toxicity and are more readily obtainable than synthetic chemicals, and plant-based substances display a variety of mechanisms of action, leading researchers to increasingly explore the developmental potential of components derived from plants for drug design during recent decades [1–3]. Psychological and behavioral disorders have become significant consequential factors associated with disability, disrupting people's emotions and moods [4]. Neuropsychiatric disorders have been identified as the third leading cause of disability, particularly among women, according to the Global Burden of Disease (GBD), Injuries, and Risk Factors Study Highlights (2017) [5]. Moreover, the World Health Organization has identified depressive disorder, which is considered the largest contributor to worldwide nonfatal health issues, with anxiety disorders representing the sixth most common contributor [6].

Depression is characterized as a multifactorial disorder that affects broad swathes of the population and is associated with physical dysfunction and increased morbidity and mortality rates, and anxiety represents another type of chronic psychiatric disorder [7]. In both developed and developing countries, gastrointestinal disorders, which are commonly associated with bacteria and parasites, represent common health issues. Enteric infections that cause dysentery-like chronic diarrhea are commonly associated with infection by numerous etiological agents, including *Salmonella*, *Campylobacter*, *Escherichia*, *Shigella*, *Yersinia enterocolitica*, parasites, and viruses [8]. Because of the development of multiple resistance against accessible drugs, current infectious diseases cannot always be readily treated; therefore, assessing plants for the presence of potential new therapeutically functional agents may represent an alternative and effective approach to the development of novel treatments.

The medicinal herb *Sterculia foetida*, which belongs to the family Malvaceae, is commonly referred to as “Jangli badam” in the Hindi and Bengali languages and as “Gorapu badam” in the Tamil language. This herb can be found in temperate and tropical regions, such as Bangladesh, North-East Australia, Malaysia, and India [9, 10]. The *S*. *foetida* tree yields edible seeds, which contain 30–36% fat and 11.4% protein, and its bark and leaves can be utilized for aperient, diuretic, and diaphoretic purposes and have also been used to treat obesity, rheumatism, gonorrhea, dropsy, and skin disease [11]. This plant also contains a wide range of bioactive components that have been isolated previously, including acetamiprid, *α*-BHC, tetradecanoic acid, methyl ester, terbufos, benfuresate, dichlofluanid, DEP (trichlorfon), 1-azuleneethanol, acetate, captan, etridiazole, diethofencarb, etobenzanid, cyfluthrin, cypermethrin, pendimethalin, kresoxim-methyl, tetraconazole, and pyributicarb [12]. Although *S*. *foetida* has broad traditional applications, several of the pharmacological characteristics of this plant remain to be explored.

Thus, the purpose of the present study was to assess the *in vitro* antidiabetic, *in vivo* neuropharmacological, and antidiarrheal activities of a methanolic extract of *S*. *foetida* (MESF). In addition, computer-aided studies, including molecular docking; absorption, disruption, metabolism, and excretion/toxicity (ADME/T); and toxicological properties of the compounds isolated from this herb were evaluated to explore their biological activities. These studies contributed to the identification of bioactive compounds present in MESF and provide insights regarding potential targets for development as lead compounds for therapeutic purposes.

## 2. Materials and Methods

### 2.1. Drugs, Chemicals, and Equipment

Phosphate buffer, potassium ferricyanide, trichloroacetic acid, ferric chloride, ascorbic acid, 2,2-diphenyl-1-picrylhydrazyl (DPPH), acarbose, and Folin–Ciocalteu reagent were all purchased from Sigma-Aldrich, St. Louis, MO, USA.

### 2.2. Plant Material Collection and Extraction

The plant material collection and detailed extraction process used to obtain the methanolic extract of *S*. *foetida* were described in detail in a previous publication [12]. The extract was stored at 4°C for additional use.

### 2.3. Ethical Statement and Experimental Animals

Swiss albino mice of both sexes, with an average weight of 20–30 grams, were obtained from Jahangirnagar University, Dhaka, Bangladesh, and were maintained under standard laboratory circumstances (room temperature: 25 ± 2°C; relative humidity: 55%–60%), with access to food and water. All experiments were performed in an isolated and soundless environment. The research was conducted in compliance with the guidelines of The Planning and Development (P&D) Committee of the International Islamic University Chittagong, Department of Pharmacy, according to current government guidelines [12].

### 2.4. *α*-Amylase Inhibitory Activity

The *α*-amylase inhibitory activity method was described in [13] with some modifications. A mixture of 1 mL of plant extract and 1 mL of *α*-amylase was incubated in a test tube at 37°C for 10 min. After preincubation, 1 mL of 1% (v/v) starch solution was added to each tube and incubated for 15 min at 37°C. After that, 0.4 mL aliquots of the incubate were transferred to sample tubes containing 3 mL starch and 2 mL phosphate buffer (pH 6.5), and 0.1 mL of the combination was removed before reincubation to measure absorbance at 565 nm; the mixture was then reincubated for 45 minutes. After mixing, 0.1 mL of the reaction mixture was removed from each tube and discharged into 10 mL of iodine solution at the conclusion of the reincubation time. The absorbance (At) was measured immediately at 565 nm after the solutions were completely mixed. The % inhibition of *α*-amylase by each plant extract can be calculated using the following formula:

(1) $$\begin{array}{c} \left[\frac{\left(\text{A0}-\text{At}\right)\text{control}-\left(\text{A0}-\text{At}\right)\text{sample}}{\left(\text{A0}-\text{At}\right)\text{control}}\right]\times 100\text{,} \end{array}$$

where A0 and At are the absorbance values at zero time and at the end of the incubation, respectively.

### 2.5. Experimental Design

For each separate behavioral test, a total of 24 mice were divided into four groups (control, standard, and two test groups), each containing six (*n* = 6) mice. The MESF was administered to the test groups at two separate doses (200 and 400 mg/kg body weight (b.w.) per os (p.o.)), whereas the control group was treated with the vehicle. For the open field test (OFT), hole cross test (HCT), thiopental sodium-induced sleeping test, elevated plus maze (EPM), and hole board test (HBT), the standard used was diazepam, whereas the standard used for the forced swim test (FST) and tail suspension test (TST) was imipramine (10 mg/kg, b.w, intraperitoneal (i.p)). In addition, loperamide (5 mg/kg) was used for both the castor oil-induced antidiarrheal test and the charcoal-induced intestinal motility test.

### 2.6. Acute Oral Toxicity Test

The acute oral toxicity test was completed according to the guidelines established for the Organization for Environmental Control Development (OECD: guidelines 420; fixed-dose method). The methods of oral toxicity test are described in previous literature [12].

### 2.7. Sedative Activity

#### 2.7.1. Open Field Test (OFT)

The open field apparatus was employed to assess locomotor activity [14]. The treatments were applied to the four groups of animals as described above in Section 2.4. The mice were placed in a box (40 cm × 40 cm × 30 cm) separated into 25 black and white squares by a series of lines on the floor. In brief, movement was recorded as the number of lines crossed by each individual mouse during a 3-minute period starting at 0, 30, 60, 90, and 120 minutes.

#### 2.7.2. Hole Cross Test (HCT)

An HCT apparatus was prepared, consisting of a cage (30 cm × 20 cm × 14 cm) separated into two chambers using a 7.5 cm tall divider featuring a hole (three centimeters in length) in the center [15]. The treatments were applied to four groups of animals, as described above in Section 2.4. The mice were placed on one side of the divider, and the number of times the mice passed through the hole during a 3-minute period was recorded, starting at 0, 30, 60, 90, and 120 minutes.

#### 2.7.3. Thiopental Sodium-Induced Sleeping Test

Treatments were applied to four groups of mice as described above in Section 2.4, and after 30 minutes, all mice were treated with thiopental sodium (40 mg/kg) to induce sleep. The latency between the administration of thiopental and the loss of the correction reflex, sleep onset, and sleep duration was recorded [16].

### 2.8. Anxiolytic Activity

#### 2.8.1. Elevated plus Maze (EPM)

The EPM utilized an apparatus situated 40 cm above the ground, featuring two open arms and two closed arms, to evaluate the anxiolytic effects of MESF in mice [17]. The arms open to a center platform, such that the maze resembles a plus sign. The treatments were applied to four groups of mice as described above in Section 2.4, and 30 minutes after treatment, each mouse was placed on the center platform, and the time spent in both the open and closed arms was recorded over a 5-minute period.

#### 2.8.2. Hole Board Test (HBT)

A device containing 16 holes (3 cm in diameter) across a flat platform was utilized in the experiment, and the apparatus was placed 25 cm above the floor [18]. The treatments were applied to four groups of mice as described above in Section 2.4, and after 30 minutes, the experimental mouse was placed on the center of the HBT device and allowed to move freely. The number of times the mouse dipped its head through the holes was recorded over a 5-minute period.

### 2.9. Antidepressant Activity

#### 2.9.1. Forced Swim Test (FST)

The glass cylinder (25 cm × 15 cm × 25 cm) was filled with water at a constant temperature of 25 ± 1°C to a height of 15 cm. The treatments were applied to four groups of animals as described above in Section 2.4, and the mice were forced to swim freely in the cylinder. The FST lasted for a total of 6 minutes, and the duration of immobility was recorded during the last 4 minutes [19].

#### 2.9.2. Tail Suspension Test (TST)

The TST test was used to assess the antidepressant activities of MESF. The treatments were applied to four groups of animals as described above in Section 2.4, after which the mice were hung by their tails to induce immobility (depression), and the time spent immobilized was recorded for six minutes [20].

### 2.10. Antidiarrheal Activity

#### 2.10.1. Castor Oil-Induced Diarrhea

Castor oil-induced diarrhea was performed as previously described [21]. Treatments were applied to four groups of animals described above in Section 2.4, and after 1 hour, 0.5 mL castor oil was administered orally. Each mouse was then placed in a box containing absorbent paper. Then, the fecal production of the mice was monitored for 4 hours for each mouse, and the absorbent paper was replaced every *h*. The defecation inhibition percentage was measured using the following equation:

(2) $$\begin{array}{c} \frac{\text{\% inhibition of defecation}=\left(\text{A}-\text{B}\right)}{\text{A}}\times 100\text{,} \end{array}$$

where *A* = average feces number of the control group and *B* = average feces number of the test group.

#### 2.10.2. Charcoal-Induced Intestinal Motility Test

The test was conducted as described previously [22]. The treatments were applied to four groups of animals as described above in Section 2.4, and after 1 hour, 0.5 mL castor oil was administered orally, followed by 1 mL of charcoal marker (10% charcoal + 5% gum acacia), 1 hour after the castor oil treatment. The animals were sacrificed 1 hour after charcoal treatment. The distance between the charcoal meal and the pylorus caecum was measured, and the overall length of the intestine (percentage) was estimated in cm. The following equation was used to determine the peristalsis index and inhibition percentage:

(3) $$\begin{array}{c} \text{\% inhibition}=\frac{\left(\text{distance travel by control}-\text{distance travel by test group}\right)}{\text{distance travel by control }}\times 100. \end{array}$$

### 2.11. Compounds Investigated

Based on the results of a previously described gas chromatography-mass spectrometry study, acetamiprid, *α*-BHC, tetradecanoic acid, methyl ester, terbufos, benfuresate, dichlofluanid, DEP (trichlorfon), 1-azuleneethanol, acetate, captan, etridiazole, diethofencarb, etobenzanid, cyfluthrin, cypermethrin, pendimethalin, kresoxim-methyl, tetraconazole, and pyributicarb were selected [12], and the chemical structures were obtained from the PubChem database.

### 2.12. *In Silico* Studies

#### 2.12.1. Molecular Docking Analysis

In this study, the ligands and proteins were prepared using the LigPrep tool and Protein Preparation Wizard, respectively, using Schrodinger Maestro v 10.1. The 3-dimensional (3D) crystallographic structures of the following proteins were obtained from the RCSB PDB [23]: human gamma-aminobutyric acid receptor (PDB : 4COF) [24], potassium channel receptor (PDB : 4UUJ) [25], human serotonin receptor (PDB : 5I6X) [26], and M3 muscarinic acetylcholine receptor (PDB ID : 4U14) [27], which were used to explore sedative, anxiolytic, antidepressant, and antidiarrheal activities, respectively.

### 2.13. ADME/T Property Analysis

SwissADME (https://www.swissadme.ch/) was used to assess each compound's pharmacokinetic properties (ADME). According to Lipinski's rule, orally active drugs must feature specific drug-likeness properties to demonstrate pharmacological reliability, such as criteria for molecular weight (MW), H-bond acceptors (HBA), H-bond donors (HBD), and partition coefficient (LogP). The admetSAR online server (https://lmmd.ecust.edu.cn/admetsar2//) was also used to predict the toxicological properties of the compounds [28].

### 2.14. Statistical Analysis

All values (*in vivo*) are described as the mean ± standard error mean (SEM). Using SPSS *v* 20.0 software, Dunnett's test was used to compare values between groups. At *p* < 0.05, 0.01, and 0.001, values were considered significant.

## 3. Results

### 3.1. *α*-Amylase Inhibitory Activity

**Figure 1:**
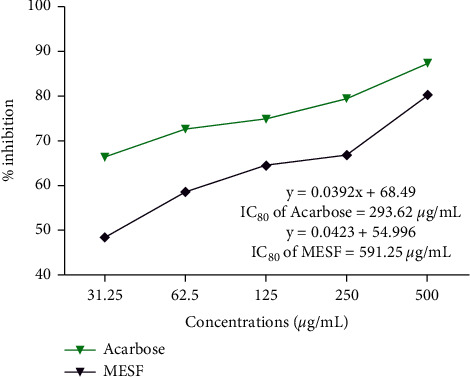
Inhibition of *α*-amylase by MESF and acarbose at various concentrations. MEAM: methanol extract of *S. foetida*.

In this test, *S*. *foetida* seeds and standard acarbose are represented as percentage of inhibition where the highest 80.28% of inhibition was achieved by 500 *μ*g/ml of MESF (Figure 1).

### 3.2. Sedative Activity

#### 3.2.1. Open Field Test (OFT)

**Figure 2:**
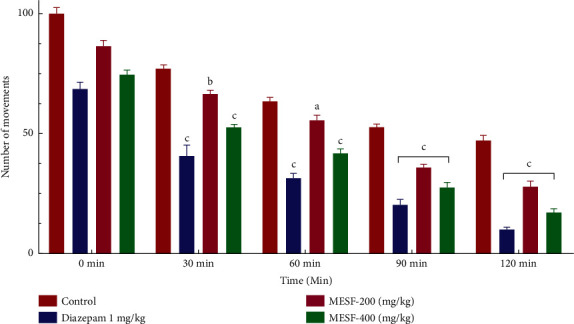
Effects of treatment with the methanolic extract of *S*. *foetida* (MESF) in mice on performance in the open field test. Values are the mean ± SEM (*n* = 6); *p* < 0.05, *p* < 0.01, and *p* < 0.001, based on Dunnett's test compared with control mice.

The present study utilized the OFT to assess the neuropharmacological activity of MESF in mice. Each mouse in the test group was treated with MESF (200 and 400 mg/kg; b.w.), which resulted in a notable decrease in the numbers of squares crossed (Figure 2). At all-time points measured in this experiment, the mice treated with the extract displayed significant decreases in locomotor activity compared with the control group.

#### 3.2.2. Hole Cross Test (HCT)

**Figure 3:**
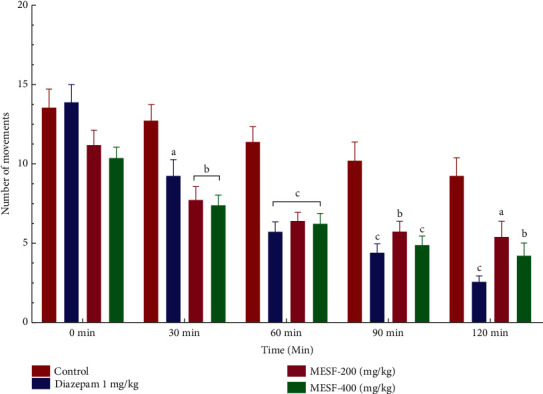
Effects of treatment with the methanolic extract of *S*. *foetida* (MESF) in mice on performance in the hole cross test. Values are the mean ± SEM (*n* = 6); *p* < 0.05, *p* < 0.01, and *p* < 0.001, based on Dunnett's test compared with control mice.

The time required for each mouse in the control group to cross from one chamber to the next ranged from 30 to 120 minutes. In this study, the groups treated with MESF demonstrated decreased locomotion at 0–120 minutes from its initial value. However, the maximal inhibition of locomotor activity was observed for both MESF doses tested (*p* < 0.001), referring to diazepam (reference drug; Figure 3).

#### 3.2.3. Thiopental Sodium-Induced Sleeping Time Test

In a dose-dependent manner, MESF (200 and 400 mg/kg) treatment resulted in a significant (*p* < 0.001) reduction in the sleep onset time, similar to that of the standard drug. In addition, the duration of sleep induced by thiopental sodium in MESF-treated animals increased compared with that of the control group (Table 1).

### 3.3. Anxiolytic Activity

#### 3.3.1. Elevated plus Maze Test (EPM)

**Figure 4:**
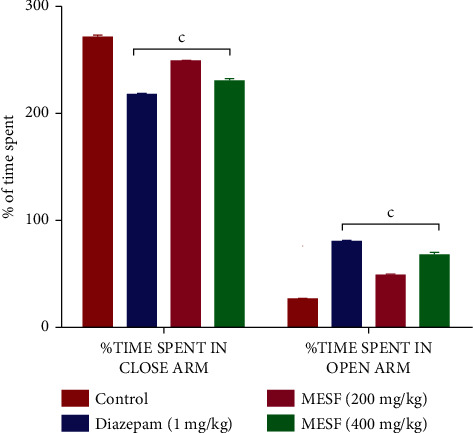
Effects of treatment with the methanolic extract of *S*. *foetida* (MESF) in mice on performance in the elevated plus maze (EPM) test. Values are the mean ± SEM (*n* = 6); *p* < 0.001, based on Dunnett's test compared with control mice.

Diazepam enhanced the amount of time spent in both the closed and open arms of the EPM substantially compared with the control mice. Mice treated with MESF at doses of 200 and 400 mg/kg showed a tendency toward spending more time in these arms. MESF-treated mice showed the greatest and most significant increase in time spent in both the closed and open arms compared with the negative control at both the 200 mg/kg and 400 mg/kg doses (*p* < 0.001 for both open and closed arms; Figure 4).

#### 3.3.2. Hole Board Test (HBT)

**Figure 5:**
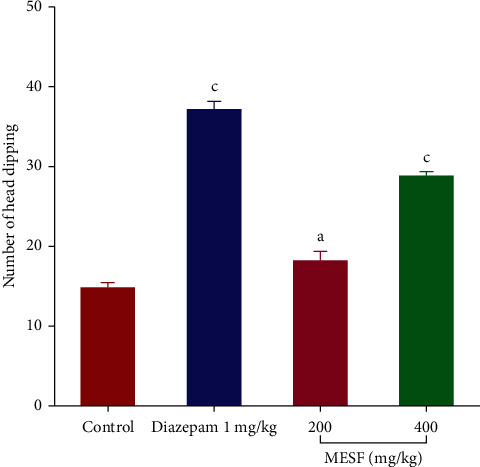
Effects of treatment with the methanolic extract of *S*. *foetida* (MESF) in mice on performance in the hole board test. Values are the mean ± SEM (*n* = 6); *p* < 0.05, *p* < 0.01, and *p* < 0.001, based on Dunnett's test compared with control mice.

MESF exhibited dose-dependent anxiolytic activity in mice, as assessed by the HBT. MESF at 400 mg/kg (*p* < 0.001) produced a greater number of head dipping events (28.83 ± 0.60) than MESF at 200 mg/kg (*p* < 0.05; 18.17 ± 0.94). Additionally, the standard drug diazepam (1 mg/kg, i.p.; *p* < 0.001) and the control group exhibited the highest (37.17 ± 0.84) and lowest numbers of head dipping events (14.83 ± 1.55; Figure 5), respectively.

### 3.4. Antidepressant Activity

#### 3.4.1. Forced Swimming Test (FST)

**Figure 6:**
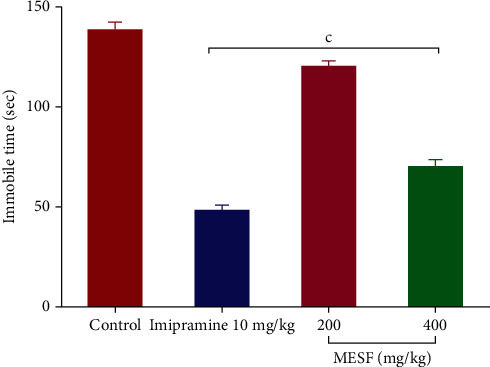
Effects of treatment with the methanolic extract of *S*. *foetida* (MESF) in mice on the performance in the forced swim test. Values are the mean ± SEM (*n* = 6); *p* < 0.001, based on Dunnett's test compared with control mice.

The animals treated with MESF (200 and 400 mg/kg) and imipramine (standard drug) revealed significant (*p* < 0.001) reduction in the period of immobility, which were measured at 120.37, 70.03, and 48.28 seconds, respectively (Figure 6) compared with the control group.

#### 3.4.2. Tail Suspension Test (TST)

**Figure 7:**
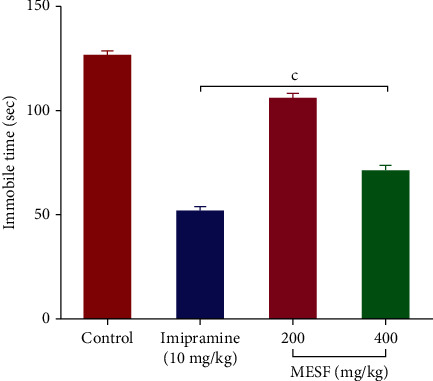
Effects of treatment with the methanolic extract of *S*. *foetida* (MESF) in mice on the performance in the tail suspension test. Values are the mean ± SEM (*n* = 6); *p* < 0.001, based on Dunnett's test compared with control mice.

MESF exhibited antidepressant-like properties in mice. Compared with the control group, which was immobile for 126.73 ± 2.0 seconds, the immobility times were 105.96 ± 2.59 seconds for 200 mg/kg and 71.25 ± 2.60 seconds for 400 mg/kg (*p* < 0.001) MESF treatment. The standard medication, however, revealed a significant (*p* < 0.001) decrease in immobility time to 51.89 ± 1.95 seconds, as shown in Figure 7.

### 3.5. Antidiarrheal Activity

#### 3.5.1. Castor Oil-Induced Diarrhea

**Figure 8:**
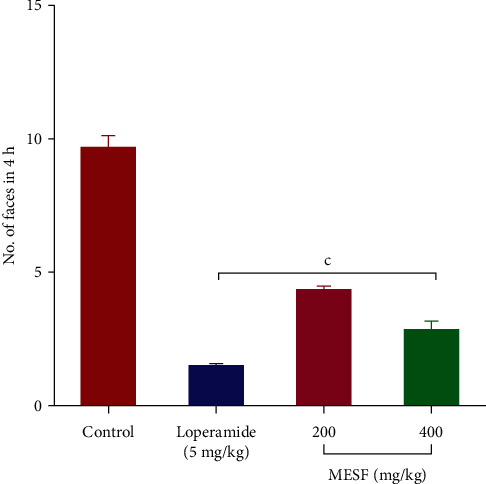
Effects of the *S*. *foetida* methanolic extract treatment on castor oil-induced diarrhea in mice. Values are the mean ± SEM (*n* = 6); *p* < 0.001, based on Dunnett's test compared with control mice.

MESF treatment induced a significant and dose-dependent inhibition of castor oil-induced defecation, as shown in Figure 8, with MESF at both 200 mg/kg inhibiting defecation by 54.94% and the dose at 400 mg/kg inhibiting defecation by 71.34%. The standard drug loperamide inhibited 84.12% of defecation.

#### 3.5.2. Charcoal-Induced Intestinal Motility Test

**Figure 9:**
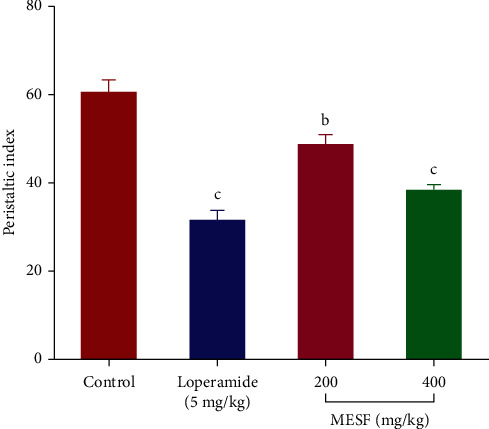
Effects of the *S*. *foetida* methanolic extract on charcoal-induced intestinal motility in mice. Values are the mean ± SEM (*n* = 6); *p* < 0.01 and *p* < 0.001, based on Dunnett's test compared with control mice.

The effects of different doses of MESF on charcoal-induced intestinal motility in mice are presented in Figure 9 as the percentage inhibition of intestinal transit. MESF (*p* < 0.001) significantly reduced the peristalsis index relative to that of the negative control group for both tested doses (*p* < 0.001). The 400 mg/kg dose had the greatest suppressive effect on intestinal motility (36.75%), similar to that of the standard drug loperamide (48.01%).

### 3.6. *In Silico* Analysis

#### 3.6.1. Molecular Docking Analysis for Sedative and Anxiolytic Properties

**Figure 10:**
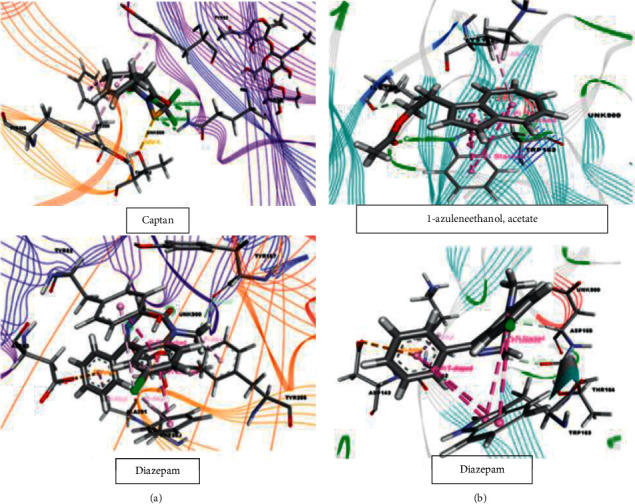
Molecular docking interactions for (a) captan and diazepam with human gamma-aminobutyric acid receptor for sedative activity (PDB ID : 4COF) and for (b) 1-azuleneethanol, acetate and diazepam with the potassium channel for anxiolytic activity (PDB ID : 4UUJ).

The 18 primary compounds identified in *S*. *foetida* were docked with the human gamma-aminobutyric acid receptor (PDB : 4COF) to test sedative effects and displayed scores between +2.296 and −6.33 kcal/mol. The results show that captan (−6.33 kcal/mol) displayed the strongest binding score with the target receptor, which was stronger than the docking score of the standard drug diazepam (−5.961 kcal/mol). The docking score ranking order is shown as follows: captan > cypermethrin > 1-azuleneethanol, acetate > dichlofluanid > DEP (trichlorfon) > etridiazole > acetamiprid > cyfluthrin > etobenzanid > benfuresate > kresoxim-methyl > *α*-BHC > tetraconazole > pendimethalin > terbufos > pyributicarb > tetradecanoic acid, methyl ester. For the anxiolytic study, our results showed that 1-azuleneethanol and acetate and tetradecanoic acid and methyl ester have the strongest and weakest binding affinities for the potassium channel (PDB : 4UUJ), with scores of −4.515 and + 1.459 kcal/mol, respectively. The reference drug diazepam displayed a docking score of −4.035 kcal/mol against the potassium channel (PDB : 4UUJ). The docking score ranking order is shown as follows: 1-azuleneethanol, acetate > DEP (trichlorfon) > dichlofluanid did not display any interactions with the target receptor. The results of the molecular docking analysis are shown in Table 2, and the ligand-target interactions are shown in Figures 10(a) and 10(b).

#### 3.6.2. Molecular Docking Study for Antidepressant Activity

**Figure 11:**
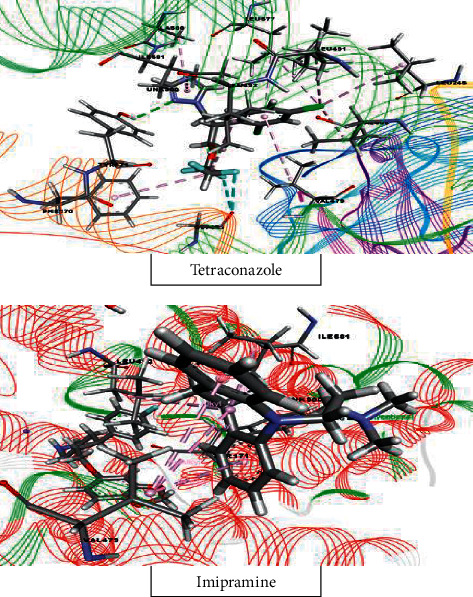
Molecular docking interaction of tetraconazole and imipramine with human serotonin receptor for antidepressant activity (PDB ID : 5I6X).

In the antidepressant docking analysis, our investigation revealed that tetraconazole and tetradecanoic acid, methyl ester have the strongest and weakest binding affinities for the human serotonin receptor (PDB : 5I6X), with docking scores of −4.842 kcal/mol and +0.842 kcal/mol, respectively. The docking score rank order for the antidepressant effect is as follows: tetraconazole > kresoxim-methyl > cyfluthrin > pyributicarb > dichlofluanid > 1-azuleneethanol, acetate > etridiazole > cypermethrin > captan > acetamiprid > diethofencarb > *α*-BHC > terbufos > pendimethalin > tetradecanoic acid, methyl ester. However, two compounds, benfuresate and DEP (trichlorfon), did not show any interactions with the target receptor. The reference standard drug imipramine displayed a docking score of −5.35 kcal/mol against the human serotonin receptor (PDB : 5I6X). These scores are shown in Table 2, and the binding interactions of the best-docked compounds are displayed in Figure 11.

#### 3.6.3. Molecular Docking Study for Antidiarrheal Activity

**Figure 12:**
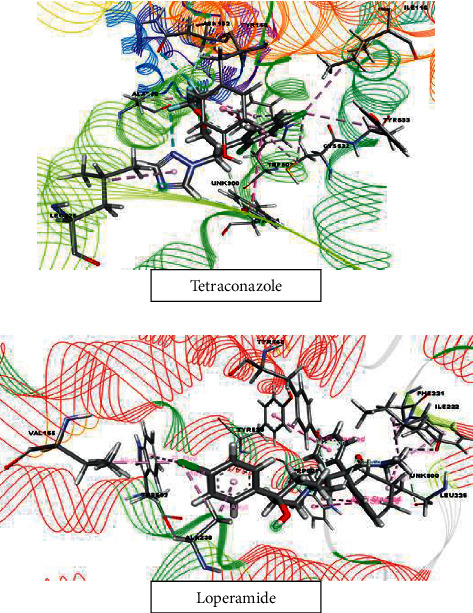
Molecular docking interaction of tetraconazole and loperamide with M3 muscarinic acetylcholine receptor for antidiarrheal activity (PDB ID : 4U14).

Tetraconazole and tetradecanoic acid, methyl ester, with docking scores of −8.106 kcal/mol and −0.749 kcal/mol, respectively, showed the strongest and weakest binding affinities against the M3 muscarinic cholinergic receptor (PDB ID : 4U14). The docking score ranking order is shown as follows: tetraconazole > etobenzanid > captan > pyributicarb > benfuresate > 1-azuleneethanol, acetate > dichlofluanid > *α*-BHC > pendimethalin > cyfluthrin > diethofencarb > acetamiprid > terbufos > kresoxim-methyl > cypermethrin > etridiazole > DEP(trichlorfon) > tetradecanoic acid, methyl ester. Loperamide (reference drug) had a binding affinity of −7.33 kcal/mol against the M3 muscarinic acetylcholine receptor (PDB ID : 4U14). All scores are shown in Table 2, and the candidate with the highest binding affinity is shown in Figure 12.

### 3.7. ADME/T and Toxicological Properties Prediction

As specified by Lipinski's rule of five, a compound may possess drug-like properties if it does not fail more than one of the following principles: MW < 500 amu; HBA sites < 10; HBD sites < 5; and lipophilicity value (LogP) ≤ 5. In addition, Veber et al. recommended that a compound should have a number of rotatable bonds ≤10 and a total polar surface area value ≤ 140 Å to provide optimal molecular flexibility, which might facilitate the transport of medications through membranes. Our study found that all tested compounds satisfied both Lipinski's rule of five and Veber's rule, which indicated that each compound could represent a good starting point for the development of new drugs (Table 3). The admetSAR online tool was used to predict the toxic properties of the compounds, and the results are presented in Table 4. None of the chemicals studied exhibited Ames toxicity, carcinogenicity, acute oral toxicity, or rat acute toxicity.

## 4. Discussion

The seeds of *S*. *foetida* were examined to assess *α*-amylase inhibitory and its behavioral effects through the application of various experimental tests, including the OFT, HCT, HBT, EPM, FST, TST, and thiopental sodium-induced sleep test, which were conducted on available and approved animal models of CNS activity.

Previous preclinical evaluation of antidiabetic study concluded that the methanol leaf extract [29], ethanol, and aqueous flower extracts of *S*. *foetida* revealed a significant reduction in diabetic rats' blood glucose levels [30]. Seeds of *S*. *foetida* are rich in secondary metabolites such as alkaloids, flavonoids, and saponins and have antidiabetic properties [12]. According to the previous study, the bioactive molecules found in the plant *S*. *foetida* play a significant role in antidiabetic properties. The current study of the antidiabetic screening test found that the methanol seed extract of *S*. *foetida* has the better antidiabetic activity.

Both the OFT and HCT were conducted to examine the effects of MESF on locomotor activity. Sedative agents will decrease locomotion, which can be assessed as a lack of inquisitive behavior when a mouse is introduced to a new environment [31]. Locomotor activity is considered to represent a mental state indicator of consciousness and awareness, and decreased locomotion serves as an indicator of sedation and a general lack of agitation, which can be interpreted as diminished CNS excitability [32]. The administration of MESF to mice reduced the number of square blocks crossed in the OFT and the number of holes crossed in the HCT starting at 30 minutes after treatment, indicating the sedative activity effects on reduced locomotion. The effects of MESF in mice were also examined using the thiopental-induced sleep test. In addition to extending the duration of sleeping time, the latent period of drowsing off is reduced and the onset of sleep in mice increased. Thiopental acts on CNS activity through the stimulation of the GABAergic system repression, and plant extracts derived from *S*. *foetida* have been shown to produce hypnosis at large dosages, such as 200 and 400 mg/kg. The extract exhibited anxiolytic-like behavior in mice during the HBT and EPM. During the HBT, anxiogenic conditions of the animals are indicated by a decrease in head dipping activity, whereas an anxiolytic state is indicated by an increase in head dipping behavior [33]. We observed that when the MESF dose increased from 200 to 400 mg/kg, the number of head dips increased, revealing the extract's anxiolytic effect. The duration spent in the open arm of the EPM test further supports the extract's anxiolytic effects.

Generally, corticotrophin-releasing factor (CRF) is produced when the hypothalamic-pituitary-adrenal (HPA) axis is overstimulated, whereas dysfunction in this system can result in the display of depressive symptoms [34]. However, potent antidepressant management suppresses the stimulation of the HPA axis caused by stress, followed by the reinstallation of CRF's rational expression and function [35]. In our study, the FST and TST were conducted following MESF administration to inspect antidepressant-like activity and the underlying mechanism of depression. These two experiments involve very stressful environments in which the mice are subjected to a state of behavioral distress, manifested as immobility, in the absence of an antidepressant agent. The administration of MESF (200 and 400 mg/kg) to mice resulted in key behaviors, including reduced immobility in the FST and an increased tendency to struggle in the TST. This antidepressant activity of MESF might be due to the inhibition of monoamine reuptake or the stimulation of HPA axis.

Diarrhea is associated with diverse recognized mechanisms, including unstable intestinal motility (motility diarrhea), infected or inflated-induced mucosal damage (exudative diarrhea), and increased water and electrolyte output (secretory diarrhea) [36]. The nitric acid found in castor oil triggers the stimulation of diarrheal activity, whereas the ricinoleic acid in castor oil triggers diarrhea via a hypersecretory response and may represent the most potent castor oil constituent [37]. In the lumen of the intestine, the castor oil containing ricinoleic acid produces ricin oleate salts with Na^+^ and K^+^, which then suppresses N^+^/K^+^ ATPase activity and consequently enhances the penetrability of the intestinal epithelium and increases water and electrolyte secretion [38, 39]. Both models for antidiarrheal activity showed the significant suppression of diarrhea activity in our study investigation. MESF (200 and 400 mg/kg) significantly suppressed defecation in the castor oil-induced diarrhea model (*p* < 0.001) and significantly inhibited intestinal motility in the charcoal-induced intestinal motility model (*p* < 0.001).

Molecular docking represents an important approach for defining ligand-target relations because it can demonstrate the conduct of minor molecules at the active sites of target proteins and can help researchers to better understand the mechanisms underlying several pharmacological reactions [22, 40]. Therefore, molecular docking was conducted to better recognize the molecular processes underlying these observed pharmacological reactions.

Eighteen phytocompounds from *S*. *foetida* were examined for sedative, anxiolytic, antidepressant, and antidiarrheal activity against four target proteins, human gamma-aminobutyric acid receptor (PDB : 4COF), potassium channel receptor (PDB : 4UUJ), human serotonin receptor (PDB : 5I6X), and M3 muscarinic acetylcholine receptor (PDB ID : 4U14), respectively.

Our findings demonstrated that three key compounds, captan, 1-azuleneethanol, acetate, and tetraconazole, docked against the target enzymes for the described pharmacological actions. The findings of our docking investigation suggested that the investigated compounds may be responsible for MESF's biological actions (sedative, anxiolytic, antidepressant, and antidiarrheal) through interactions with the target proteins.

In addition, the ADME/T analysis demonstrated that captan, 1-azuleneethanol, acetate, and tetraconazole meet Lipinski's rule of five, and these compounds did not possess any toxic properties, including Ames toxicity, carcinogenicity, acute oral, or rat acute toxicity (Tables 3 and 4). Drugs or compounds that violate these rules are not considered to demonstrate good oral bioavailability [41, 42].

## 5. Conclusions

In summary, various phytochemicals, which may induce synergistic or individual effects, are responsible for such pharmacological activities. These chemical compounds may offer such pharmacological activities as the molecular docking study assessed. In addition, the *in silico* docking analysis indicated that captan, 1-azuleneethanol, acetate, and tetraconazole might represent suitable candidates for sedative, anxiolytic, antidepressant, and antidiarrheal activities. However, further widespread research remains necessary to inspect the complete array of biological reactions and mechanisms in animal models. Additional advanced studies remain to identify the potential compounds responsible for pharmacological activities.

## Figures and Tables

**Table 1:** Effects of treatment with the methanolic extract of *S*. *foetida* (MESF) in mice on thiopental sodium-induced sleeping time.

Group	Dose (mg/kg)	Onset of sleep (min)	Duration of sleep (min)
Control	-	18.82 ± 1.44	31.97 ± 4.08
Diazepam	1	6.61 ± 0.70^c^	176.19 ± 2.49^c^
MESF	200	9.81 ± 0.99^c^	156.24 ± 6.24^c^
MESF	400	7.72 ± 0.88^c^	169.93 ± 7.41^c^

Values are the mean ± SEM (*n* = 6); ^c^*p* < 0.001, based on Dunnett's test compared with control mice.

**Table 2:** Molecular docking analysis for the major bioactive compounds.

Compounds	Docking score (kcal/mol)			
	Sedative (4COF)	Anxiolytic (4UUJ)	Antidepression (5I6X)	Diarrhea (4U14)
Acetamiprid	−4.694	−2.917	−3.937	−5.912
Alpha-BHC	−3.957	−3.556	−3.804	−6.678
Tetradecanoic acid, methyl ester	+2.296	+1.459	+0.842	−0.749
Terbufos	−3.416	−2.895	−3.500	−5.847
Benfuresate	−4.432	−4.263	—	−7.297
Dichlofluanid	−4.987	—	−4.716	−6.908
DEP (trichlorfon)	−4.899	−4.278	—	−5.124
1-azuleneethanol, acetate	−5.537	−4.515	−4.616	−7.239
Captan	−6.330	−3.265	−3.959	−7.549
Etridiazole	−4.828	−3.758	−4.266	−5.428
Diethofencarb	−4.841	−3.828	−3.860	−6.239
Etobenzanid	−4.620	−3.744	−4.377	−7.674
Cyfluthrin	−4.647	−3.941	−4.821	−6.585
Cypermethrin	−5.694	−2.718	−4.150	−5.771
Pendimethalin	−3.794	−4.069	−3.434	−6.677
Kresoxim-methyl	−4.235	−3.115	−4.834	−5.776
Tetraconazole	−3.805	−4.217	−4.842	−8.106
Pyributicarb	−3.104	−3.652	−4.815	−7.502
Standard (diazepam/imipramine/loperamide)	−5.961	−4.035	−5.35	−7.33

**Table 3:** Physicochemical properties of the compounds, demonstrating good oral bioavailability.

Compound	MW	HBA	HBD	LogP	AMR	nRB	TPSA	Lipinski's violations
Rule	<500	<5	≤10	≤5	40–130	≤10	≤140	≤1
Acetamiprid	222.67 g/mol	3	0	1.64	59.77	3	52.28 Å^2^	0
Alpha-BHC	290.8 g/mol	0	0	3.39	57.62	0	0	0
Tetradecanoic acid, methyl ester	242.4 g/mol	2	0	4.81	75.50	13	26.30 Å^2^	0
Terbufos	288.4 g/mol	2	0	3.72	78.34	8	110.96 Å^2^	0
Benfuresate	256.32 g/mol	4	0	2.51	65.55	3	60.98 Å^2^	0
Dichlofluanid	333.2 g/mol	4	0	2.73	74.59	5	74.30 Å^2^	0
DEP (trichlorfon)	257.43 g/mol	4	1	1.10	47.76	4	65.57 Å^2^	0
1-azuleneethanol, acetate	214.26 g/mol	2	0	2.95	63.74	4	26.30 Å^2^	0
Captan	300.6 g/mol	2	0	2.26	69.90	2	62.68 Å^2^	0
Etridiazole	247.5 g/mol	3	0	2.62	50.45	3	63.25 Å^2^	0
Diethofencarb	267.32 g/mol	4	1	2.75	74.44	8	56.79 Å^2^	0
Etobenzanid	340.2 g/mol	3	1	4.03	87.87	7	47.56 Å^2^	0
Cyfluthrin	434.3 g/mol	0	5	5.22	108.92	7	59.32 Å^2^	0
Cypermethrin	416.3 g/mol	4	0	4.96	108.97	7	59.32 Å^2^	0
Pendimethalin	281.31 g/mol	4	1	2.41	82.55	6	103.67 Å^2^	0
Kresoxim-methyl	313.3 g/mol	5	0	3.23	87.83	7	57.12 Å^2^	0
Tetraconazole	372.14 g/mol	7	0	3.87	76.35	7	39.94 Å^2^	0
Pyributicarb	330.4 g/mol	3	0	4.09	98.01	6	66.68 Å^2^	0

MW = molecular weight (g/mol); HBA = hydrogen bond acceptor; HBD = hydrogen bond donor; LogP = lipophilicity; AMR = molar refractivity; nRB = number of rotatable bonds; TPSA = topological polar surface area.

**Table 4:** Toxicological properties of the selected bioactive secondary metabolites in MESF.

Compound	Parameters			
	Ames toxicity	Carcinogens	Acute oral	Rat acute
Captan	Ames toxic	Noncarcinogens	III	1.9636
1-azuleneethanol, acetate	Ames toxic	Noncarcinogens	III	1.8435
Tetraconazole	Non-Ames toxic	Noncarcinogens	III	2.5785

Category-III (500 mg/kg < LD_50_ < 5,000 mg/kg).

## Data Availability

Available data are presented in the article.

## Abbreviations

b.w.: Body weight
d.w.: Distilled water
i.p.: Intraperitoneally
PASS: Prediction of activity spectra for substances
ADME/T: Absorption, distribution, metabolism, excretion, or toxicity.
